# Knowledge, attitudes and practices of meat hygiene among slaughterhouse workers and retail meat sellers in Bangladesh

**DOI:** 10.1016/j.heliyon.2024.e40066

**Published:** 2024-11-01

**Authors:** Md Asibul Hasan, Md Bashir Uddin, Syed Sayeem Uddin Ahmed

**Affiliations:** a Department of Epidemiology and Public Health, Sylhet Agricultural University, Sylhet, 3100, Bangladesh; b Department of Medicine, Sylhet Agricultural University, Sylhet, 3100, Bangladesh

**Keywords:** Meat hygiene, Knowledge, Attitudes, Practices (KAP), Slaughterhouse, Meat handlers, Bangladesh

## Abstract

Inadequate handling of raw meat leading to cross-contamination, often stemming from insufficient knowledge and practices among meat handlers, poses a significant global health challenge, especially in developing nations where food-borne diseases are prevalent. Effective understanding and implementation of preventive measures by meat handlers are essential in reducing the incidence of food-borne illnesses and the contamination of raw meat. This study aimed to assess the knowledge, attitudes, and practices regarding meat hygiene among retail meat sellers and slaughterhouse personnel in the Bogura district of Bangladesh. A descriptive, cross-sectional survey was conducted, encompassing 408 meat handlers selected through a multistage sampling approach. Data on their knowledge, attitudes, and practices of meat hygiene were collected using a structured questionnaire, and then analyzed. The participants had a mean age of 34.63 years with a standard deviation of 13.44. The majority of participants demonstrated good knowledge (70.70 %) and positive attitudes (53 %). However, no significant differences were observed in good (49.80 %) or poor (50.20 %) practices regarding meat hygiene. Interestingly, significant associations were observed between knowledge and age (*p* = 0.022), location (*p* < 0.001), and borderline significance in training (*p* = 0.051). Similarly, a statistically significant association was found between the practice of meat hygiene and age (*p* = 0.041), level of education (*p* = 0.008), and training (*p* = 0.004). Furthermore, a significant association existed between knowledge and practices of meat hygiene (*p* < 0.001). Older meat handlers with training exhibited better knowledge and practices of meat hygiene compared to their younger counterparts. This study underscores the importance of public health education, policy development, and regular training and retraining programs for meat handlers to ensure safe meat handling practices and overall hygiene.

## 1 Introduction

Foodborne illnesses pose a significant public health challenge, particularly in developing countries, notably in the Indian subcontinent, due to inadequate food handling practices and sanitation measures. Critical factors contributing to this issue include insufficient food safety regulations, flawed regulatory frameworks, limited financing for the procurement of safer equipment, and inadequate training for food handlers [1]. The true burden of foodborne illnesses remains underestimated due to insufficient data, compounded by the fact that many individuals affected do not seek medical attention. These illnesses, ranging from diarrheal diseases to various forms of cancer and, in severe cases, death, affect societies globally [2].

Animal-derived foods pose a significant risk to human health unless proper food hygiene measures are implemented. Meat, in particular, has been identified as a major source of foodborne illnesses. Despite changes in production and processing systems, surveillance studies continue to highlight the persistence of pathogens such as *Salmonella* spp., *Yersinia enterocolitica*, and *Escherichia coli* in meat [2]. However, there remains a lack of comprehensive understanding regarding the risk posed by bacterial illnesses transmitted through the consumption of meat and animal products [3].

Efforts to address food safety issues have been insufficient despite guidance provided by WHO-FAO and the Codex Alimentarius Commission (CAC) [4]. To enhance awareness and promote food safety principles, the WHO-FAO General Assembly designated June 7th as World Food Safety Day, starting from 2019 [5].

According to the World Health Organization (WHO), foodborne illnesses result in significant morbidity and mortality worldwide, particularly affecting children under the age of five, with Africa and Southeast Asia bearing the greatest burden [6]. These illnesses not only cause personal suffering but also strain healthcare systems, hinder socioeconomic growth, and pose challenges to foreign trade and tourism.

In Bangladesh, the livestock industry significantly influences development and economic progress, generating employment opportunities and contributing to GDP [7]. However, challenges in ensuring meat safety throughout the supply chain persist, particularly in rural abattoirs and developing countries where meat assessors lack the necessary expertise and resources [8].

Proper assessment of live animals before slaughter and stringent inspection procedures during the slaughtering process are essential to ensure the safety of meat consumed by the public. However, many slaughterhouses in developing countries lack adequate equipment, clean water sources, waste disposal systems, and refrigeration units, leading to contaminated meat and improper waste disposal [9].

In Bangladesh, the knowledge, attitudes, and practices (KAP) of food handlers regarding food safety and foodborne diseases (FBDs) play a crucial role in the promotion of food safety and the protection of human health against FBDs. There is a lack of comprehensive research on the KAP of meat handlers regarding food hygiene, despite the increasing consumption of meat domestically and internationally [10]. Evidence from domestic or local research findings helps policy makers to adopt accurate measures to address health issues that affect public health. Therefore, evaluation of meat handlers in Bogura district of Bangladesh, is necessary to address the increasing incidence of foodborne illnesses and formulate effective policies to improve meat hygiene and public health. In this study, we aim to assess the knowledge, attitudes and practices regarding meat hygiene among retail meat vendors and slaughterhouse personnel in Bogura district of Bangladesh.

## 2 Materials and methods

### 2.1 Study area

**Fig. 1:**
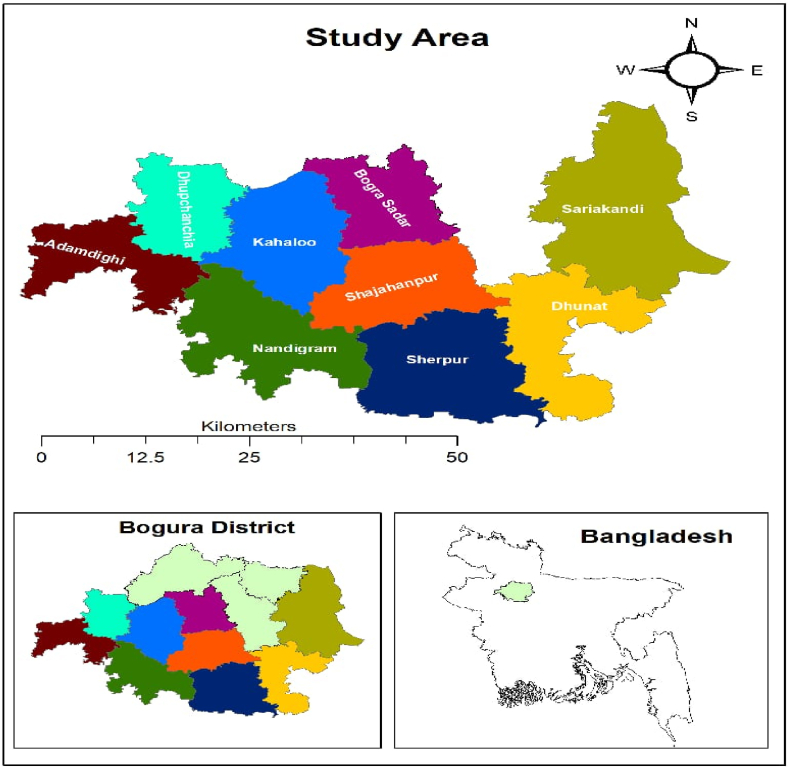
Study area of Bogura District, Bangladesh.

The study was conducted across 68 slaughterhouses and meat shops spanning nine upazilas in Bogura district (Fig. 1), situated at the heart of the North Bengal region. Positioned within the Rajshahi division, the city is bounded by the Korotoa and Jamuna rivers (latitude: 24° 51′ 0″ N, longitude: 89° 22′ 0″ E). This area was selected owing to its major input into the meat business, with a notable demand for meat obtained from indigenous varieties of cattle, sheep, goats, and buffalo. Moreover, offal consumption is a prevalent custom among the local community, with kidneys, hearts, livers, bone marrow, head muscles, and intestines being the most commonly consumed offal items. The nine upazilas were selected using a multistage sampling approach to ensure comprehensive coverage of the region. These upazilas were chosen based on their population density, economic importance in the meat trade**,** and accessibility. They represent a cross-section of both urban and rural settings within the district, providing a broader understanding of meat hygiene practices across different socioeconomic and infrastructural environments. The 68 slaughterhouses and meat selling shops were selected based on their size, operational capacity, and geographical distribution across the nine upazilas, covering the major slaughterhouses and meat-selling shops in the region. Among the 68 business establishments, 39 were meat selling shops and 17 were slaughterhouses, while 12 served both purposes.

### 2.2 Study population

The study included individuals working in slaughterhouses and those selling meat, encompassing both slaughterhouse workers and meat vendors across Bogura district, Bangladesh.

### 2.3 Study design

A cross-sectional study was employed to assess the knowledge, attitudes, and practices related to meat hygiene among meat vendors and slaughterhouse workers in both abattoirs and meat shops in Bogura. Information regarding the target population's knowledge, attitudes, and practices concerning meat hygiene was gathered using face-to-face questionnaires and key informant interviews.

### 2.4 Sample size determination

In this context, the formula [11] was adapted for estimating the prevalence of meat hygiene knowledge, attitudes, and practices (KAP) rather than a specific disease. A prevalence value of 50 % was assumed due to the lack of prior studies on the KAP of meat hygiene in this population. This assumption ensures a conservative sample size that provides adequate power for the study.

$$n=Z\alpha^{2}\mathrm{PQ}/L^{2}$$

where,

**n** = is the appropriate sample size.

**Zα** = the normal deviate that provide 95 per cent confidence interval (1.96).

**P** = a prior estimate of the prevalence of disease. (A prevalence value of 50 % was assumed due to the lack of previous study).

**Q** = 1-p.

**L** = the allowable error

$$n=1.96^{2}\times \left(0.5\times 0.5\right)/\;\left(0.05\right)$$

The total minimum sample size is 384.

### 2.5 Data collection

The study used a single structured questionnaire for data collection, which was administered to all respondents. The questionnaire was pre-tested before data collection to ensure its reliability and validity. The development of this questionnaire was guided by comprehensive literature on food safety [[12], [13], [14]]. This was structured into four sections in alignment with the study objectives. The initial section focused on capturing demographic information about the respondents. Respondents were selected using a multistage sampling approach, targeting meat sellers and slaughterhouse workers across nine upazilas in Bogura district. Specific selection criteria, such as working history of 3 months or more, and an age ≥14 years (here referred to as “Children”) are the two main eligibility criteria for becoming a respondent. The subsequent sections delved into assessing their knowledge level, attitudes, and personal hygiene practices within slaughterhouses and meat shops. We have used a Kish Grid method for selecting the respondents to be interviewed.

The questionnaire format varied, with some questions employing a binary ‘Yes’ or ‘No’ scale, while others utilized a three-point Likert scale, ranging from 1 = strongly agree to 3 = strongly disagree. Clear instructions regarding the purpose of the questionnaire and how to complete it were provided.

To ensure confidentiality, each questionnaire was assigned an identification number instead of using individuals' names. The administration of the questionnaires was conducted by the researcher.

### 2.6 Scoring and grading

The assessment of meat hygiene knowledge consisted of seventeen questions, with one point awarded for each correct answer and zero points for incorrect responses. A total score above 8 indicated a good level of knowledge, while a score of 8 or lower indicated poor knowledge [15,16].

Twenty-two statements regarding meat handlers' opinions and attitudes towards meat hygiene were presented, with responses rated on a scale of agreement (1), indifference (0), and disagreement (2). Scores ranged from 22 to 44, and were summed and averaged. The overall mean score was 31.5 ± 4.97 standard deviation.

Attitudes were classified into three categories: a mean score of 34–44 denoted a good attitudes, 34–30 indicated a fair attitudes, and scores below 30 suggested a poor attitudes.

Additionally, twenty-four questions regarding workplace practices concerning meat hygiene were posed. Each correct response earned one point, while incorrect answers received zero points. A total score exceeding 15 indicated good practice, whereas a score of 15 or lower indicated poor practice.

### 2.7 Data analysis

Data collected from meat shops and slaughterhouses was entered into the Microsoft Excel sheet and then cleaned. Statistical analysis involved examining proportions, means, and standard deviations. Cross-tabulations were generated to compare categorical data using Chi-square tests and *p*-values for continuous variables. A *p* < 0.05 indicated statistical significance, and the analysis was conducted using SPSS software. Additionally, correlation and association between KAP regarding meat hygiene were determined using a correlation matrix and chi-square tests, respectively. Inferential statistics, including chi-square tests, were utilized in testing the study hypothesis.

### 2.8 Ethical approval

The research was carried out in compliance with the protocols and standards, and received authorization from the Ethics Committee at Sylhet Agricultural University, Bangladesh (Protocol #ARP2023046). The participants were informed about the purpose and procedures and voluntary consent were taken. Here, employing children in slaughterhouses and meat-selling shops presents both ethical and legal challenges. While it is acknowledged that children occasionally work in informal sectors in Bangladesh, their participation was included with proper assent, considering the local context. Many countries, including Bangladesh, have implemented laws that prohibit child labors in hazardous industries, such as slaughterhouses. However, enforcement of these laws poses a significant challenge in many areas or regions. Contributing factors include poverty, limited access to education, and weak regulatory systems, which all enabled the continued presence of child labor in health hazardous sectors like meat processing. Therefore, it is crucial to enforce existing laws that protect children, raise awareness of the risks associated with child labor in slaughterhouses, and strengthen regulatory mechanisms to ensure compliance.

## 3 Results

### 3.1 The respondents’ demographic information

Among the 437 meat handlers who were approached, 408 participants consented to partake in the research, yielding a notable response rate of 93.30 %. The demographic attributes of the participants are presented in Table 1. The mean age of the participants’ was 34.63 years old, with a standard deviation of 13.44 years. A majority of the participants were young, comprising 54 % of the total. Sherpur and Dhonut upazilas collectively represented the highest proportion of respondents at 15 % each. The vast majority (89 %) had achieved at least a primary level of education, and the majority (68 %) were involved in meat selling. Approximately half (52 %) had received training from authorities.

**Table 1:** Socio demographic characteristics of respondents.

Characteristics	Frequency	percentage
**Study Area**	26	6 %
Adomdighi	26	6 %
Bogra sadar	40	10 %
Dhonut	63	15 %
Dupcacia	37	9 %
Kahalu	42	10 %
Nondigram	47	12 %
Sariakandi	58	14 %
Shahajanpur	33	8 %
Sherpur	62	15 %
Total	408	100 %
**Occupation**		
Meat Seller	276	68 %
Slaughter house worker	132	32 %
Total	408	100 %
**Age**		
Old	101	25 %
Middle Aged	73	18 %
Young	220	54 %
Children	14	3 %
**Education**		
Graduate^a^	18	4 %
High-Secondary	8	2 %
Secondary	97	24 %
Primary	239	59 %
Illiterate	46	11 %
Total	408	100 %
**Training**		
Yes	213	52 %
No	195	48 %
Total	408	100 %

a Graduate refers a “Bachelor's degree or equivalent”.

### 3.2 Knowledge assessment

#### 3.2.1 Knowledge of meat hygiene among meat traders and slaughterhouse workers

The analysis of respondents' knowledge and their corresponding responses is presented in Table 2. A large majority of respondents (81 %) recognized that meat contamination poses a significant risk due to its limited shelf life. However, approximately 86 % of respondents were unaware that handlers with diarrheal syndromes could pose a risk, and only 14 % of respondents were knowledgeable about the sources of meat contamination**.**

**Table 2:** Knowledge of respondents.

Variables	Frequency	N%
**Can meat spoilage be caused by microorganisms?**		
NO	145	36 %
YES	263	64 %
**Is the contamination of meat very risky due to the shelf life?**		
NO	76	19 %
YES	331	81 %
**Could unsanitary practices be a source of carcass contamination?**		
NO	179	44 %
YES	229	56 %
**Can a handler with diarrheal syndromes be a source of risk?**		
NO	351	86 %
YES	57	14 %
**Can water be a source of microbial contamination?**		
NO	92	23 %
YES	316	77 %
**Can water from hoses used for cleaning be a source of contamination of carcasses?**		
NO	275	67 %
YES	133	33 %
**Heard about meat borne disease?**		
NO	299	73 %
YES	109	27 %
**Know the Reason for carcass contamination?**		
NO	109	27 %
YES	299	73 %
**Does Contamination pose any health risk to meat consumers?**		
NO	174	43 %
YES	234	57 %
**Regular washing of hands reduces the risk of meat?**		
NO	124	30 %
YES	284	70 %
**Using appropriate gloves& Mask reduces contamination?**		
NO	144	35 %
YES	264	65 %
**Meat inspection to rule out infection is important?**		
NO	311	76 %
YES	97	24 %
**Cleanliness of the facility is important for meat processing facility?**		
NO	85	21 %
YES	323	79 %
**Washing of live animals is important before slaughter?**		
NO	241	59 %
yes	167	41 %
**The clean and dirty part of meat should be processed separately?**		
NO	263	64 %
YES	145	36 %
**Proper knowledge of potential contamination sources?**		
NO	350	86 %
YES	58	14 %
**Touching of hair after hand wash can cause contamination?**		
NO	320	78 %
YES	88	22 %
**Should keep short nails?**		
NO	146	36 %
YES	262	64 %

#### 3.2.2 Overall knowledge assessment

**Fig. 2:**
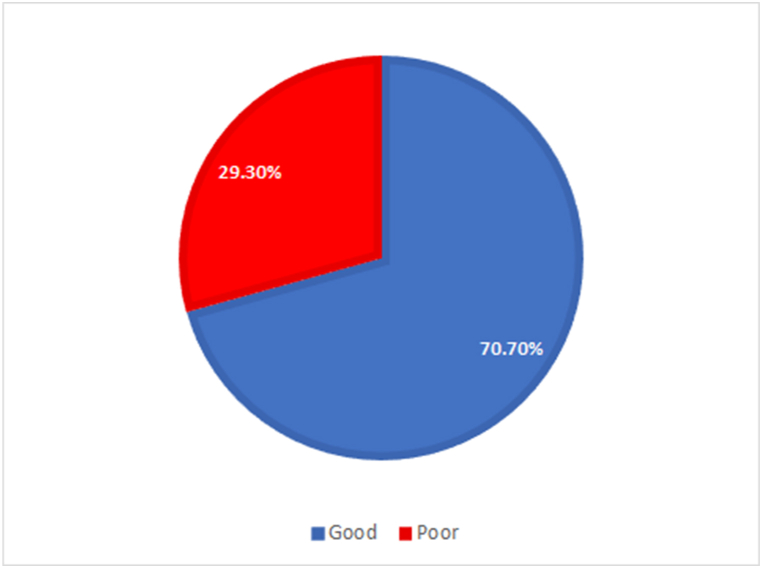
Overall knowledge of meat hygiene assessment.

The overall knowledge scores of the study participants are depicted in Fig. 2. The majority of meat handlers, including both meat sellers and slaughterhouse workers (70.70 %) demonstrated good knowledge, while 29.30 % exhibited a poor understanding of meat hygiene.

#### 3.2.3 Assessment of knowledge of meat hygiene on basis of demographic factors

Further, the knowledge of meat hygiene was measured based on various demographic factors (Table 3). The mean knowledge score of 9.14 represents the average level of knowledge among all respondents, while the standard deviation of 2.069 indicates the variation in knowledge among them. Among the upazilas of Bogura, respondents from Kahalu have the highest average knowledge score of 10.40, while respondents from Nondigram upazila exhibit the highest variation in their knowledge level, with a standard deviation of 2.383. The low *p*-value of 0.000 indicates a statistically significant difference in knowledge among different locations. Regarding occupation, meat sellers and slaughterhouse workers have an average knowledge scores of 9.25 and 8.92, respectively, with standard deviations of 2.172 and 1.823. The *p*-value of 0.137 suggests that there may not be a significant difference between the two occupations. In terms of age, the mean knowledge scores for children, young adults, aged adults, and older adults are 7.28, 8.79, 9.18, and 9.52, respectively. The *p*-value of 0.020 indicates a significant difference in knowledge among age categories. Regarding education level, respondents with a high secondary education have the highest average knowledge score of 9.50, while illiterate respondents have the lowest. The overall average knowledge level among respondents is 9.17, with a standard deviation of 2.069. The *p*-value of 0.097 suggests that there may not be a significant difference between different education levels. Furthermore, respondents who received training have a higher mean knowledge score of 9.35, compared to those who did not receive training, who have a slightly lower average score of 8.95. Although there appears to be a difference in mean scores between the two groups, the borderline *p*-value of 0.051 indicates that this difference may not be statistically significant at the conventional threshold of 0.05.

**Table 3:** Knowledge assessment on basis of demography.

Variables	Mean	Std. Deviation	*p*-value
**Location**			0.000
Adomdighi	9.54	2.177	
Bogra Sadar	9.13	2.355	
Dhonut	8.70	1.613	
Dupcacia	9.73	2.130	
Kahalu	10.40	2.037	
Nondigram	9.40	2.383	
Sariakandi	9.03	2.224	
Shahajanpur	8.00	1.000	
Sherpur	8.76	1.743	
Total	9.14	2.069	
**Occupation**			0.137
Meat seller	9.25	2.172	
Slaughter house worker	8.92	1.823	
Total	9.14	2.069	
**Age**			0.020
Old	9.52	2.262	
Middle aged	9.18	1.897	
Young	8.79	2.056	
children	7.28	1.936	
Total	9.14	2.069	
**Education**			0.097
Graduate^a^	9.20	1.757	
Higher Secondary	9.50	2.268	
Secondary	9.00	1.947	
Primary	9.30	2.146	
Illiterate	8.83	1.959	
Total	9.17	2.069	
**Training**			0.041
Yes	9.35	2.109	
No	7.95	2.018	
Total	8.65	2.069	

a Graduate refers a “Bachelor's degree or equivalent”.

#### 3.2.4 Attitudes towards meat hygiene practices

The attitudes of respondents towards meat hygiene are shown in Table 4. A significant majority agreed that it is their responsibility to handle meat safely (94 %), refrain from selling spoiled meat to customers (80 %), and maintain short nails (70 %). Moreover, most of them also concurred that cleaning equipment before slaughter is advisable (73 %), though only 23 % found it easy to maintain cleanliness in their working environment. The data reveals a lack of information among respondents, with nearly half (49 %) lacking attitudes about meat safety, which is crucial for personal well-being. Additionally, 28 % did not possess adequate attitudes about using gloves when handling meat, and 51 % were unaware of the importance of wearing aprons during work. Furthermore, a majority of respondents disagreed on several topics, including the necessity of using gloves during meat handling (44 %), the mandatory requirement of hand washing with disinfectant after toilet use (44 %), the necessity of wearing protective equipment such as an apron (44 %), the importance of ante-mortem and post-mortem meat inspection for hygienic meat production (41 %), and whether knives need changing or sterilization after each processing (61 %).

**Table 4:** Respondents attitudes towards meat hygiene.

Variables	Frequency	N%
**It is a responsibility to handle meat safely**		
Agree	384	94 %
No idea	24	6 %
**Trained is necessary for safe meat handling**		
Agree	283	69 %
Disagree	107	26 %
No idea	21	5 %
**Is Meat safety critical for personal health?**		
No idea	201	49 %
Agree	137	34 %
Disagree	70	17 %
**Does it matter if use gloves or not during handling meat?**		
No idea	113	28 %
Agree	117	29 %
Disagree	178	44 %
**Should not sell spoiled meat to customers**		
No idea	15	4 %
Agree	328	80 %
Disagree	67	16 %
**Workers should keep their nails small**		
Agree	284	70 %
Disagree	124	30 %
**Meat should only handle by workers who do not have cut nails or injured fingers**		
Agree	295	72 %
Disagree	113	28 %
**Diarrhea cannot prevent one from selling meat**		
No idea	57	14 %
Agree	157	38 %
Disagree	194	48 %
**Hand washing after the toilet with a disinfectant is mandatory**		
No idea	34	8 %
Agree	196	48 %
Disagree	178	44 %
**Disinfecting abattoir premises is a way to avoid contamination**		
No idea	104	25 %
Agree	243	60 %
Disagree	61	15 %
**The wearing of protective equipment (Apron) is necessary**		
No idea	208	51 %
Agree	22	5 %
Disagree	178	44 %
**Cleaning the slaughter area before slaughter operations**		
No idea	7	2 %
Agree	278	68 %
Disagree	124	30 %
**Cleaning of equipment before slaughter is desirable**		
No idea	8	2 %
Agree	297	73 %
Disagree	104	25 %
**The deposit of organ meats on the ground is prohibited**		
No idea	130	32 %
Agree	278	68 %
**Eating and drinking in the slaughter area should be disallowed**		
No idea	227	56 %
Agree	95	23 %
Disagree	86	21 %
**Ante mortem and post-mortem meat inspection is essential to hygienic meat production**		
No idea	102	25 %
Agree	136	33 %
Disagree	168	41 %
**Rubbing of meat with fresh blood to make it look good should be discouraged as it reduces good hygiene in meat processing**		
Agree	273	67 %
Disagree	135	33 %
**There is need to change or sterilize your knives after each processing**		
No idea	114	28 %
Agree	44	11 %
Disagree	250	61 %
**In this job, it is more important to work quickly than keep the carcasses clean**		
Agree	113	28 %
Disagree	295	72 %
**This type of working environment, keeping clean is easy**		
Agree	95	23 %
Disagree	313	77 %
**A small amount of dirt on clothing or utensils will not cause any harm**		
No idea	48	12 %
Agree	211	52 %
Disagree	149	37 %
**If meat is well-cooked then it is always safe to eat**		
No idea	37	9 %
Agree	251	62 %
Disagree	117	29 %

#### 3.2.5 Overall attitudes assessment

**Fig. 3:**
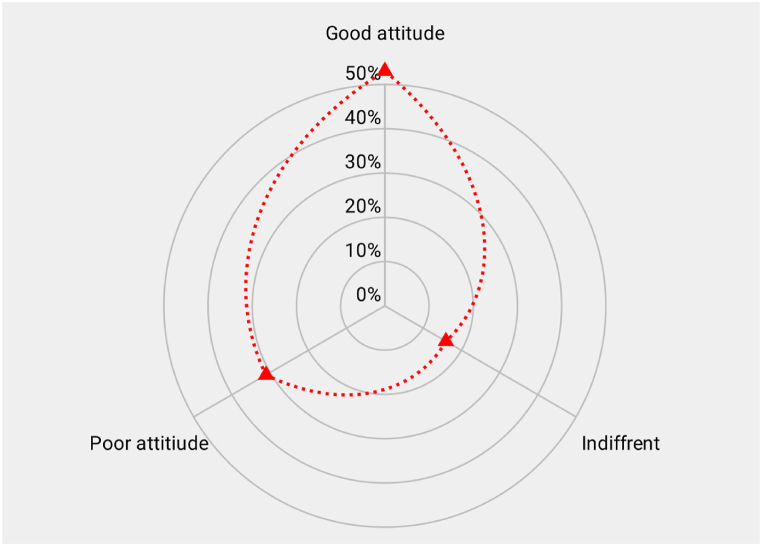
Overall attitudes of meat hygiene.

Fig. 3 depicts the overall attitudes of respondents towards meat hygiene. Approximately, 53 % of the respondents demonstrated a positive attitudes towards meat hygiene, while 16 % showed indifference, and 31 % displayed a negative attitudes towards meat hygiene.

#### 3.2.6 Assessment of attitudes towards meat hygiene on basis of demographic factors

According to Table 5, the mean value of 32.76 represents the average attitudes towards meat hygiene among all respondents, while the standard deviation of 4.979 indicates the variation in attitudes towards meat hygiene among them. Among the Upazilas of Bogura, respondents from Kahalu have the highest average attitude towards meat hygiene (32.50), while respondents from Nondigram Upazila exhibit the highest variation in attitudes towards meat hygiene, with a standard deviation of 5.225. The *p*-value of 0.476 suggests a statistically non-significant difference in attitudes towards meat hygiene across different locations. In terms of occupations, meat sellers and slaughterhouse workers have average attitudes towards meat hygiene scores of 32.81 and 32.67 respectively, with standard deviations of 5.196 and 4.507. The *p*-value of 0.589 indicates that there might not be a significant difference in attitudes towards meat hygiene between the two occupations.

**Table 5:** Attitudes assessment towards meat hygiene on basis of demography.

Variables	Mean	Std. Deviation	*p*-value
**Location**			0.476
Adomdighi	33.65	5.253	
Bogra Sadar	33.20	5.667	
Dhonut	32.83	5.034	
Dupcacia	33.76	5.535	
Kahalu	32.50	3.218	
Nondigram	33.30	5.225	
Sariakandi	32.22	5.235	
Shahajanpur	31.94	4.703	
Sherpur	32.16	4.754	
Total	32.76	4.979	
**Occupation**			0.589
Meat seller	32.81	5.196	
Slaughter house workers	32.67	4.507	
Total	32.76	4.979	
**Age**			0.031
Old	32.96	4.397	
Middle Aged	33.73	5.017	
Young	32.76	4.979	
Children	31.44	5.126	
**Education**			0.43
Graduate^a^	32.56	1.822	
Higher Secondary	34.00	4.243	
Secondary	32.20	6.246	
Primary	33.09	4.536	
Illiterate	32.13	5.110	
Total	32.76	4.979	
**Training**			0.070
Yes	33.09	5.113	
No	32.40	4.814	
Total	32.76	4.979	

a Graduate refers a “Bachelor's degree or equivalent”.

In age categories, the mean attitudes scores for children, young individuals, adults, and older respondents are 31.44, 32.76, 33.73, and 32.96 respectively, with standard deviations of 5.126, 4.979, 5.017, and 4.397. The *p*-value of 0.031 indicates a significant difference in attitudes towards meat hygiene among the age groups. Among the respondents, those with higher secondary education have the highest average attitudes towards meat hygiene score of 34.00, while illiterate respondents have the lowest attitudes level of 32.13. The overall average attitudes towards meat hygiene among respondents is 32.76, with a standard deviation of 4.979. The *p*-value of 0.043 suggests a significant difference in attitudes towards meat hygiene among respondents with different education levels.

Regarding training, respondents who received training have a mean score of 33.09 with a standard deviation of 5.113, while those who did not receive training have a lower mean score of 32.40 and a slightly lower standard deviation of 4.814. Although there is a slight difference in mean scores between the two groups, the *p*-value of 0.070 indicates that this difference may not be statistically significant at the conventional threshold of 0.05.

#### 3.2.7 Practices of meat hygiene

The perspectives of respondents regarding meat hygiene practices during the slaughter, processing, and selling phases were assessed (Table 6). The data indicates that while a majority (61 %) of individuals wash their hands before handling meat, a concerning low percentage (5 %) use soap or detergent powder during hand washing. Furthermore, only 75 % of respondents claim to wash their hands every time after using the restroom. Encouragingly, a higher proportion (91 %) ensure the use of enough clean water during meat processing. However, there are still areas of potential risk, as evidenced by practices such as handling carcasses when sick or suffering from diarrheal syndromes (69 %) and the placement of cutters and winches on the floor (60 %). Additionally, despite the majority (95 %) refrigerating unsold meat after processing. There is a significant gap in using personal protective equipment (PPE), with only 30 % using face masks and 14 % using caps during the processing and selling stages.

**Table 6:** Respondents practices assessment.

Variables	Frequency	N%
**Do you wash your hand before handling meat?**		
NO	159	39 %
YES	249	61 %
**If so, do you wash them with one of the soaps/detergent powder?**		
NO	387	95 %
YES	21	5 %
**Do you wash your hands every time you use the restroom?**		
YES	307	75 %
NO	103	25 %
**Washing hand With or without soap?**		
NO	288	71 %
YES	120	29 %
**Do you handle carcasses when you have injuries on your hands?**		
NO	321	79 %
YES	87	21 %
**Do you handle carcasses when you are sick or suffering from diarrhea syndromes?**		
NO	126	31 %
YES	282	69 %
**Do you keep your fingernails long?**		
NO	345	85 %
YES	63	15 %
**Do you wear gloves during slaughter?**		
NO	358	88 %
yes	50	12 %
**During slaughter, is there any contact with the skin, walls, floor, or equipment?**		
YES	291	71 %
NO	107	26 %
**Count of Do you use an apron during the process?**		
NO	374	92 %
Yes	34	8 %
**Do you use boots during slaughter?**		
NO	290	71 %
YES	118	29 %
**Do you place the cutters and winches on the floor?**		
NO	164	40 %
YES	244	60 %
**Are carcasses and offal placed in direct contact with floors, walls, or other equipment during hide removal and transport operations?**		
NO	228	56 %
YES	180	44 %
**Do you clean slaughter equipment daily?**		
YES	282	69 %
NO	126	31 %
**Do you clean the area before slaughter or selling?**		
YES	346	85 %
No	62	15 %
**Do you clean the area after processing or selling?**		
NO	183	45 %
YES	225	55 %
**Do you Clean the front knives?**		
YES	349	86 %
NO	59	14 %
**Do you process carcass and intestine together in the same place?**		
YES	304	75 %
NO	104	25 %
**Do you use enough clean water to process your meat?**		
YES	371	91 %
NO	37	9 %
**Do you wash the animals before slaughtering?**		
NO	148	36 %
YES	260	64 %
**Do you rub meat with blood after processing to make it look fresh?**		
NO	340	83 %
YES	68	17 %
**Do you refrigerate your meat after processing if remain unsold?**		
NO	21	5 %
YES	387	95 %
**Do you use face mask during the process & selling?**		
NO	287	70 %
YES	121	30 %
**Do you use Cap during the process & selling?**		
NO	349	86 %
Yes	59	14 %

#### 3.2.8 Overall practices of meat hygiene

**Fig. 4:**
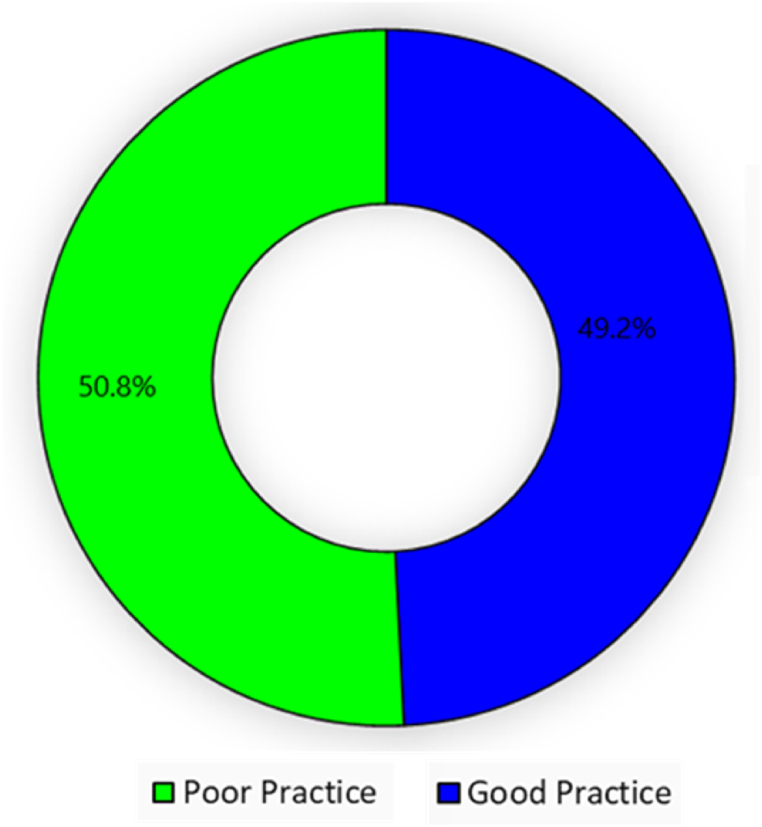
Overall meat hygiene practices.

The respondents' overall practices in relation to meat hygiene is illustrated in Fig. 4. Notably, 49.2 % of respondents exhibited scores >15 which are considered good practices, while 50.8 % who scored ≤15 demonstrated behaviors categorized as poor practices.

#### 3.2.9 Assessment of practices of meat hygiene on basis of demographic factors

Based on the data provided in Table 7, the mean value of 13.27 reflects the average level of meat hygiene practices among all respondents, with a standard deviation of 1.693 indicating variability in these practices among them. Notably, Bogura Sadar stands out among the Upazilas of Bogura with both the highest average practices score (13.07) and the greatest variability (2.147) in meat hygiene practices. The *p*-value of 0.468 suggests no statistically significant difference in the practices of meat hygiene across various locations.

**Table 7:** Assessment of practices of meat hygiene on basis of demographic characters.

Variables	Mean	Std. Deviation	*p*-value
**Location**			0.468
Adomdighi	13.42	1.793	
Bogra Sadar	13.58	2.147	
Dhonut	13.41	1.700	
Dupcacia	13.51	1.592	
Kahalu	13.07	1.068	
Nondigram	13.36	1.762	
Sariakandi	13.17	1.865	
Shahajanpur	12.70	1.571	
Sherpur	13.16	1.549	
Total	13.27	1.693	
**Occupation**			0.522
Meat seller	13.30	1.698	
Slaughter house worker	13.19	1.686	
Total	13.27	1.693	
**Age**			0.041
Old	13.21	1.630	
Middle Aged	13.73	1.264	
Young	12.76	2.017	
Children	10.92	2.173	
Total	12.66	1.771	
**Education**			0.008
Graduate^a^	12.06	2.363	
Higher Secondary	14.50	0.926	
Secondary	13.32	1.840	
Primary	13.28	1.610	
Illiterate	13.33	1.367	
Total	13.27	1.693	
**Training**			0.004
Yes	13.46	2.571	
No	12.05	1.796	
Total	12.75	2.183	

a Graduate refers a “Bachelor's degree or equivalent”.

When examining occupations, meat sellers and slaughterhouse workers show similar average practices scores of 13.30 and 13.19, respectively, with no significant difference in practices between these occupations. Regarding age categories, middle-aged respondents have the highest mean practices score (13.73), followed by old (13.21) and young (12.76) individuals, with a significant difference in practices among age groups reflected by a *p*-value of 0.041. In terms of education, those with higher secondary education demonstrate the highest average practices (13.33), indicating variations across educational levels. Illiterate respondents have the lowest score (14.50) in meat hygiene practices. The *p*-value of 0.008 suggests a significant difference in practices across educational levels. Regarding training, respondents who received training have a higher mean practices score (13.46) compared to those who did not (12.05), and the *p*-value of 0.004 indicates a significant difference in meat hygiene practices between these groups.

### 3.3 Measurers of dependency among knowledge attitudes & practices regarding meat hygiene

#### 3.3.1 Association between KAP of meat hygiene

The relationship between the practices of meat hygiene by individuals handling meat and their knowledge and attitudes towards meat hygiene is presented in Table 8. A significant association was found between the knowledge of meat hygiene and practices by meat sellers and slaughterhouse workers. The results of the chi-square (χ^2^) analysis indicate significant associations between knowledge and attitudes categories, highlighting a strong relationship between these variables. In the knowledge category, individuals categorized as having good knowledge significantly outnumbered those with poor knowledge, with a substantial chi-square value of 127.88 (*p* < 0.0001). This suggests a significant association between knowledge levels and the categorical outcomes of good and poor. Similarly, in the attitude domain, a statistically significant association was observed, as individuals with poor attitudes significantly outnumbered those with fair and good attitudes, yielding a chi-square value of 40.13 (*p* < 0.0001).

**Table 8:** Association between knowledge, attitudes and practices of meat hygiene.

Variables	Good Practices (N)	Poor Practices (N)	Total(N)	Statistics (χ^2^)	*p*-value
**Knowledge**					
Good	231	47	278	127.8815387	<0.0001
Poor	33	96	129		
**Attitudes**					
Poor	26	38	217	40.13349618	<0.0001
Fair	76	50	126		
Good	174	43	64		

#### 3.3.2 Correlation among KAP concerning meat hygiene

The correlation matrix presented in Table 9 elucidates the connections among knowledge, attitudes, and practices regarding meat hygiene among meat sellers and slaughterhouse workers in the Bogura district. A robust positive correlation of 0.8306508 between knowledge and attitudes suggests that individuals with higher levels of knowledge generally hold more favorable attitudes toward meat hygiene practices. Similarly, the correlation coefficient of 0.6834276 between knowledge and practices indicates a moderately strong positive correlation, indicating that individuals with greater knowledge about meat hygiene tend to demonstrate better hygiene practices. Additionally, the correlation coefficient of 0.5828168 between attitudes and practices suggests a moderate positive correlation, implying that individuals with more positive attitudes toward meat hygiene are likely to engage in better practices. Overall, these correlations underscore the interconnectedness of knowledge, attitudes, and practices in the realm of meat hygiene among meat sellers and slaughterhouse workers, indicating that heightened knowledge and positive attitudes often correspond with improved hygiene practices in this specific demographic.

**Table 9:** Correlation matrix among knowledge, attitudes, and practices concerning meat hygiene.

Category	Knowledge	Attitudes	Practices
Knowledge	1.0000000	0.8306508	0.6834276
Attitudes	0.8306508	1.0000000	0.5828168
Practices	0.6834276	0.5828168	1.0000000

## 4 Discussion

The knowledge, attitudes, and practices (KAP) among slaughterhouse workers and retail meat sellers in Bangladesh reveal significant gaps that can influence public health. Socio-demographic factors such as education, training and occupation play crucial roles in shaping these KAP outcomes.

In this study, the demographic data collected from slaughterhouse workers and retail meat sellers in Bogura District, Bangladesh, presents a diverse representation of the study population., Regarding education, the primary level predominates (59 %), indicating the need for targeted educational interventions. The proportion of respondents with basic primary education is slightly higher than the findings from Jigjiga, where 52.7 % of participants had received at least basic primary education [17]. The percentage of individuals attending secondary school in Bogura city was 24 %, which is nearly half of the 42.9 % of respondents working with meat in Khulna city [16]. This congruence in educational levels may be attributed to Bogura's metropolitan nature and high population density. The level of education observed in this study aligns closely with findings from Kano but is lower compared to other studies [[17], [18], [19]]. A majority of respondents identify their occupation as meat sellers (68 %). Interestingly, more than half of the participants have undergone some form of training (52 %), suggesting a willingness to engage with educational initiatives [16,20]. This study reinforces these findings, revealing a significant correlation between knowledge and the practical application of meat hygiene among meat sellers and slaughterhouse workers. The age distribution, spanning children, young, middle-aged, and old individuals, presents a varied demographic profile, with a notable concentration in the young category (54 %). The mean age of respondents in this study (34.63 years ±13.44 SD) closely resembles findings from previous studies, highlighting the importance of training and educational programs [20] aimed at enhancing meat hygiene practices among slaughterhouse workers and retail meat sellers in Bogura District, Bangladesh.

The responses from meat sellers and slaughterhouse workers provide a comprehensive overview of variables related to meat spoilage, contamination risks, and preventive measures within meat shops and slaughterhouses. The data highlights that microorganisms could be a significant cause of meat spoilage, with 64 % of respondents acknowledging this risk, a finding supported by researchers [2,21]. Contamination from various sources, including unsanitary practices and handlers with diarrheal syndromes, poses substantial risks, as recognized by the majority of respondents. Additionally, water, especially from cleaning hoses, emerges as a prevalent source of microbial contamination, a finding similar to that noted in the earlier study [17]. Awareness of meat-borne diseases is somewhat lacking, as 73 % claim they are unheard of, indicating gaps in knowledge. Nevertheless, there is recognition of the importance of preventive measures such as hand washing, glove, and mask usage, as well as facility cleanliness, consistent with the findings from Akabanda et al. [22]. Overall, 70.70 % of respondents demonstrate good knowledge of meat hygiene, a finding similar to that observed in this study. However, gaps exist in knowledge regarding contamination sources and proper handling procedures, as indicated by the high percentage of respondents lacking awareness in these areas. Frequent professional training could help improve knowledge among slaughterhouse workers and meat sellers, mitigating contamination risks and ensuring consumer safety. Statistical analysis revealed a significant association between age and knowledge of meat hygiene, with older respondents exhibiting superior knowledge compared to their younger counterparts, consistent with the previous findings [23]. However, in contrast to a study conducted in Ibadan, Southwest Nigeria, where younger meat handlers showed a greater understanding of food hygiene, our study found older respondents to have better knowledge [24]. A statistically significant difference was also found between the knowledge levels of trained and untrained personnel, with trained respondents demonstrating better knowledge of meat hygiene. Training for meat handlers can enhance their knowledge and safety practices, benefiting both themselves and others, particularly newcomers with limited knowledge of meat hygiene [25].

The study findings present a nuanced perspective, highlighting both strengths and areas requiring improvement in meat handling and safety protocols. The majority of respondents (94 %) acknowledged their responsibility to handle meat safely, indicating commendable awareness of personal accountability for meat product safety. Additionally, a significant proportion (80 %) recognized the importance of refraining from selling spoiled meat to customers, demonstrating a commitment to consumer well-being.

However, the study also identified areas of concern regarding knowledge and adherence to meat hygiene practices. Nearly half of the respondents (49 %) lacked knowledge about meat safety, emphasizing the need for comprehensive education and training programs. Similarly, a considerable proportion (51 %) were unaware of the importance of wearing aprons during work, indicating gaps in understanding basic hygiene measures. Divergent views on crucial aspects of meat handling and safety were observed, with a notable proportion of respondents disagreeing on the necessity of using gloves during meat handling (44 %) and the importance of ante-mortem and post-mortem meat inspection for hygienic meat production (41 %). Such disparities underscore the need for standardized protocols and increased awareness regarding best practices in meat hygiene. Furthermore, discrepancies in opinions regarding certain practices were noted. For example, while a majority agreed on the necessity of cleaning equipment before slaughter (73 %), a significant portion (23 %) found maintaining cleanliness in their working environment challenging, suggesting practical barriers hindering effective implementation of hygiene measures. The respondents' attitudes towards meat hygiene were also lacking in some aspects, as several of them disagreed that it should be forbidden to smear blood on meat to make it look fresh. This finding aligns with similar attitudes observed in other studies [10,17]. Regarding attitudes, slaughterhouse workers demonstrated a moderate level of concern for meat hygiene, with an average attitudes score of 32.67, while meat sellers exhibited a slightly higher average score of 32.81, indicating a marginally more favorable attitudes. The standard deviation of 4.507 for slaughterhouse workers suggests a relatively consistent attitudes, whereas meat sellers showed greater variability with a standard deviation of 5.196. The non-significant *p*-value (0.589) implies that any observed difference in average attitudes scores between meat sellers and slaughterhouse workers may be due to random variation and might not be statistically meaningful. Understanding the nuances in attitudes among different occupational groups can inform targeted strategies to enhance awareness and compliance with hygiene standards, ensuring the overall safety of meat products in the region [26,27]. Further research exploring factors influencing attitudes towards meat hygiene in these groups could provide a more comprehensive understanding of the dynamics at play.

This study highlights both commendable practices and areas of concern that require urgent attention and improvement. While a majority (61 %) of respondents do wash their hands before handling meat, the fact that only 5 % use soap or detergent powder during hand washing is concerning. Effective hand washing with soap is crucial in removing harmful bacteria and pathogens, reducing the risk of contamination during meat handling. Additionally, while 75 % of respondents claim to wash their hands after using the restroom, this figure should ideally be higher to maintain proper hygiene standards. Consistent hand hygiene is imperative in preventing the spread of diseases and ensuring consumer safety [28].

Furthermore, the data highlights certain risk practices, such as handling carcasses when sick or suffering from diarrheal syndromes (69 %). This poses a significant threat to public health, increasing the likelihood of foodborne illnesses and meat contamination. The low utilization of personal protective equipment (PPE) is also alarming, with only 30 % of respondents using face masks and 14 % using caps during meat processing and selling. Proper PPE usage is vital in preventing the transmission of pathogens and ensuring the safety of workers and consumers. Moreover, around 60 % of respondents place cutters and winches on the floor, increasing the risk of contamination and compromising meat quality. However, the majority (95 %) refrigerate unsold meat after processing, helping to preserve freshness and reduce the growth of harmful bacteria. Statistical analysis revealed a significant difference in practice levels between trained and untrained personnel, with trained respondents demonstrating better practice of meat hygiene. Additionally, there was a significant difference in practices among various age groups, with respondents in the middle age group (31–45) showing better practices. This observation is supported by previous studies [29,30], which found that older and trained meat handlers tended to practices appropriate meat hygiene more than younger and untrained respondents.

Though the study findings are interesting, there are a few limitations. The small sample size, while suitable for initial analysis, might impede interpreting the KAP findings at large. In addition, the execution of the research, limited to one district, confines the various factors that could influence KAP in meat hygiene.

## 5 Conclusion

In summary, the data show that respondents had good meat hygiene knowledge and practices, but their attitudes and practices seem to differ. In particular, the report emphasizes the need for slaughterhouse hygiene improvements. It is worth noting that education and training initiatives demonstrate superior knowledge and practices. Overall, respondents' knowledge and attitudes toward meat hygiene are associated with their practices of meat hygiene. Addressing gaps in knowledge, promoting proper hygiene practices, and providing adequate training can help improve meat hygiene and ensure public health safety. Although these findings are interesting for the country, further micro-level research is required that takes into account other dimensions of meat hygiene and public health.

## CRediT authorship contribution statement

**Md Asibul Hasan:** Writing – review & editing, Writing – original draft, Methodology, Investigation, Formal analysis, Data curation, Software, Validation, Visualization. **Md Bashir Uddin:** Writing – review & editing, Writing – original draft, Supervision, Resources, Project administration, Methodology, Investigation, Data curation, Conceptualization, Software, Validation, Visualization. **Syed Sayeem Uddin Ahmed:** Writing – review & editing, Writing – original draft, Visualization, Validation, Supervision, Software, Resources, Project administration, Methodology, Investigation, Funding acquisition, Formal analysis, Data curation, Conceptualization.

## Data availability

The data that support the findings of this study are included in the text.

## Funding

No funding was received to conduct this study.

## Declaration of competing interest

The authors declare that they have no known competing financial interests or personal relationships that could have appeared to influence the work reported in this article.

## References

[1] Aluko O.O., Ojeremi T.T., Olaleke D.A., Ajidagba E.B. (Jun. 2014). Evaluation of food safety and sanitary practices among food vendors at car parks in Ile Ife, southwestern Nigeria. Food Control.

[2] Park M.S., Kim Y.S., Lee S.H., Kim S.H., Park K.H., Bahk G.J. (Mar. 2015). Estimating the burden of foodborne disease, South Korea, 2008-2012. Foodb. Pathog. Dis..

[3] Lianou A., Panagou E.Z., Nychas G.J.E. (May 2017). Meat safety—I foodborne pathogens and other biological issues. Lawrieś Meat Sci..

[4] FAO and WHO (2023). https://openknowledge.fao.org/handle/20.500.14283/cc5042en.

[5] FAO and WHO (2023). A guide to World food safety day. https://openknowledge.fao.org/handle/20.500.14283/cc3926en.

[6] World Bank. “Food Safety Handbook : A Practical Guide for Building a Robust Food Safety Management System (English). Washington, D.C. : World Bank Group. http://documents.worldbank.org/curated/en/450921587054767474/Food-Safety-Handbook-A-Practical-Guide-for-Building-a-Robust-Food-Safety-Management-System.

[7] (2020). Ministry of Fisheries and Livestock. https://www.scribd.com/document/463073930/G-1-09-144-Fisheries-English-pdf.

[8] Codex Alimentarius code of hygiene practice for meat CAC/RCP 58-2005. http://www.fao.org/downloads/standards.

[9] Daniel Lucas, Prudencio, Adriana Márcia, Nicolau Korres, Álex Pires Carneiro (2024). Sustainability indicators in beef slaughterhouses: a systematic review. Desarrollo Local Sostenible.

[10] Kehinde G., Adejimi A., Abiola A.-H. (2020). Assessment of knowledge, attitude, and practice of meat hygiene among meat handlers in Lagos State, Nigeria. Niger. J. Gen. Pract..

[11] Pourhoseingholi M.A., Vahedi M., Rahimzadeh M. (2013). Sample size calculation in medical studies. Gastroenterol. Hepatol. From Bed to Bench.

[12] Al Banna M.H., Disu T.R., Kundu S. (2021). Factors associated with food safety knowledge and practices among meat handlers in Bangladesh: a cross-sectional study. Environ. Health Prev. Med..

[13] Laxman Ghimire, Santosh Dhakal YR., Pandeya S., Chaulagain B.R., Mahato R.C., Satyal, Dinesh Kumar, Singh (2013). Assessment of pork handlers' knowledge and hygienic status of pig meat shops of Chitwan district focusing campylobacteriosis risk factors. Int. J. Infect..

[14] Abduelrahmana M.Y., Adama S.Y., Ahmed A.A., Eltahir H.A. (2024). Knowledge, attitude, and practice of meat hygiene among butchers in abattoirs and meat markets in Wadi Salih Garsila, Central Darfur - Sudan. Am. J. Zool..

[15] Zemachu Ashuro, Nathnael Zeysse, Mulugeta Ayalew (2023). Meat hygiene knowledge, handling practices and associated factors among meat handlers in Gedeo zone, Ethiopia. Dental Sci. Rep..

[16] Biswas G., S Md, Islam S., M M., Rahman Md, Islam M. (2024).

[17] Tegegne H.A., Phyo H.W.W. (2017). Food safety knowledge, attitude and practices of meat handler in abattoir and retail meat shops of Jigjiga Town, Ethiopia. J. Prev. Med. Hyg..

[18] Jianu C., Goleţ I. (Aug. 2014). Knowledge of food safety and hygiene and personal hygiene practices among meat handlers operating in western Romania. Food Control.

[19] Firdaus Siau M., Son R., Mohhiddin O., Toh P.S., Chai L.C. (2015). Food court hygiene assessment and food safety knowledge, attitudes and practices of food handlers in Putrajaya. Int. Food Res. J..

[20] Md Jisan, Ahmed, Mahabbat Ali, Khorshed Alam, Mohammad Shameem, Al Mamun M.K.A., Bhuiyan, Priyanka Bhandari, Roshan Chalise S.M., Zannatul Naem, Manju Rahi, Khairul Islam, Fahmida Bristy, Amina Khatun, Mirza Synthia Sabrin (2024).

[21] Erkmen O., Bozoglu T.F. (2016).

[22] Akabanda F., Hlortsi E.H., Owusu-Kwarteng J. (Jan. 2017). Food safety knowledge, attitudes and practices of institutional food-handlers in Ghana. BMC Publ. Health.

[23] Olumakaiye M.F., Bakare K.O. (2013). Training of food providers for improved environmental conditions of food service outlets in urban area Nigeria. Food Nutr. Sci..

[24] Kehinde Gbolabo J., Adejimi Adebola Afolake, Abiola Abdul-Hakeem O. (Jul–Dec 2020). Assessment of knowledge, attitude, and practice of meat hygiene among meat handlers in Lagos State, Nigeria. Niger. J. Gen. Pract..

[25] Aslam M., Malik M., Kausar S. (2022). Effect of food safety and hygiene training on KAP score among food handlers in multiple food service institution, Pakistan. J. Food Safe Hyg..

[26] Adekunle Lawrence Bello, Usman Oladipo, Adekanye, Ochuko Orakpoghenor, Talatu Patience, Markus (2023). Knowledge, attitude and practices of abattoir workers and veterinarians toward meat safety in abattoir or slaughter slabs within Uyo Metropolis, Akwa Ibom State, Nigeria. J. Health Sci. Res..

[27] Victoria Kimindu, Dasel W.M., Kaindi, Lucy Gicuku, Njue, Samwel Maina, Githigia (2023). Meat safety knowledge, attitude and practices of slaughterhouse workers in Kajiado, Kenya. Vet. Med. Sci..

[28] Vidya Rao (2024). The effectiveness of hand hygiene in preventing zoonotic diseases among zoo workers: a critical evaluation and strategic recommendations. EPRA Int. J. Multidiscip. Res..

[29] Rabindra Bhandari, Anil Kumar, Singh, Prakash Raj, Bhatt, Ashish Timalsina R., Bhandari, Pratibha Thapa, Jijeebisha Baral, Sunil Adhikari, Pramila Poudel, Sudip Chiluwal, Prakash C., Joshi Nabin, Bahadur Adhikari (2022). Factors associated with meat hygiene-practices among meat-handlers in Metropolitan City of Kathmandu, Nepal. PLOS Glob. Public Health.

[30] Grace Lamunu, Christopher Ddamulira, Florence Ajok, Odoch, Paul Katamba, David R Mutekanga (2022). Factors affecting adherence to meat hygiene practices of beef butcheries in Kasangati Town Council, Wakiso District, Uganda. World J. Adv. Res. Rev..

